# Voluntary wheel running exercise improves sleep disorder, circadian rhythm disturbance, and neuropathology in an animal model of Alzheimer's disease

**DOI:** 10.1002/alz.70314

**Published:** 2025-06-24

**Authors:** Yiying Hu, Long Niu, Yixin Chen, Huijia Yang, Xinhui Qiu, Fei Jiang, Cong Liu, Huaibin Cai, Weidong Le

**Affiliations:** ^1^ Key Laboratory of Liaoning Province for Research on the Pathogenic Mechanisms of Neurological Diseases The First Affiliated Hospital of Dalian Medical University Dalian China; ^2^ Department of Neurology The First Affiliated Hospital of Dalian Medical University Dalian China; ^3^ Department of Neurology Heping Hospital affiliated to Changzhi Medical College Changzhi China; ^4^ Dalian Seventh People's Hospital Dalian China; ^5^ Interdisciplinary Research Center on Biology and Chemistry Shanghai Institute of Organic Chemistry Chinese Academy of Sciences Shanghai China; ^6^ Transgenic Section, Laboratory of Neurogenetics National Institute on Aging National Institutes of Health Bethesda Maryland USA; ^7^ Center for Clinical and Translational Medicine Shanghai University of Medicine and Health Sciences Shanghai China; ^8^ Center for Clinical Research on Neurological Diseases the First Affiliated Hospital Dalian Medical University Dalian China

**Keywords:** Alzheimer's disease, circadian rhythm, sleep–wake cycle, suprachiasmatic nucleus, voluntary wheel running exercise

## Abstract

**INTRODUCTION:**

The sleep–wake cycle and circadian rhythm disturbances are common in Alzheimer's disease (AD). However, it is not known if exercise has any benefit for the sleep disorders in AD.

**METHODS:**

We conducted a 2‐month voluntary wheel running (VWR) exercise (Ex) in an animal model of AD (APP^SWE^/PS1^dE9^ mice). We assessed behavioral circadian rhythm, sleep structure, circadian clock genes, cognitive function, and neurodegeneration in the suprachiasmatic nucleus (SCN), the hippocampus, and the cortex.

**RESULTS:**

After VWR exercise in the AD mice, the rapid eye movement sleep was increased by 89%. The levels of circadian clock genes were significantly changed (brain and muscle arnt‐like protein 1 [BMAL1] and retinoic acid receptor‐related orphan receptorsα [RORα] reduced by 45.7% and 36.4%, reverse erythroblastosis virusα (REV‐ERBα) increased by 119%) in the SCN by immunofluorescence staining, with the mRNA levels were markedly altered (*Bmal1* and *Rorα* decreased by 57% and 68%, *Rev‐erbα* elevated by 79%) in the hypothalamus at Zeitgeber Time 1; phospho‐tau 231 (p‐tau231) was reduced by 35%, whereas vesicular GABA transporter (VGAT) was elevated by 38.7% in the SCN. In addition, ionized calcium binding adapter molecude 1 (Iba1), glial fibrillary acidic protein (GFAP), amyloid β (Aβ), and p‐tau231 were significantly reduced in the hippocampus and cortex.

**DISCUSSION:**

Our results demonstrate that VWR exercise improves sleep disorders, cognitive deficits, and neuropathology in AD mice.

**Highlights:**

- Voluntary wheel running (VWR) exercise improves the behavioral circadian rhythm disorder and sleep structure disturbance in Alzheimer's disease (AD) mice.
- After VWR exercise, there is a significant change in the expression levels of circadian clock genes, and a remarkable reduction of tau phosphorylation and axonal damage in the γ‐aminobutyric acid (GABA)ergic neurons in the suprachiasmatic nucleus (SCN).
- The levels of beta‐site amyloid precurson protein cleaving enzyme 1 (BACE1) and glycogen synthase kinase‐3β (GSK3β) are reduced in the hypothalamus after VWR exercise in AD mice.
- Furthermore, VWR exercise attenuates cognitive deficits, neuroinflammation, amyloid beta (Aβ), and phospho‐tau protein accumulation in the hippocampus and cortex.

## 1 BACKGROUND

Alzheimer's disease (AD) is the most common cause of dementia, with an expensive burden in this century.^1^ Its pathologic processes include amyloid beta (Aβ) deposition, tau protein phosphorylation, and neurodegeneration in the hippocampus, cortex, and other brain regions.^2^ Advanced age and heritable factors are the serious risks of AD.^3^ Aside from cognitive impairment, 80% of patients also display neuropsychiatric symptoms, including sleep abnormality.^4^ Patients with AD often experience circadian rhythm sleep–wake disorders, difficulties in falling or maintaining asleep, nocturnal awakenings, and excessive daytime sleepiness.^5^ The sleep architecture change in patients with AD shows decreased total sleep time, rapid eye movement (REM) sleep percentage, and slow‐wave sleep, but the wake stages shift is increased with fragmented sleep.^6^ Sleep disturbance and its associated circadian rhythm disorder is a major risk factor for the progression of AD, which may precede earlier in the onset of memory problems by decades and worsen with the cognitive impairment advance.^7^ This sleep–wake cycle is regulated by the suprachiasmatic nuclei (SCN) of the hypothalamus, the primary circadian pacemaker with γ‐aminobutyric acid (GABA)ergic neurons.^8^ Meanwhile, GABAergic neurons may play a critical role in the pathogenesis of AD.^9^

Circadian clock genes drive a circadian rhythm with a day–night cycle in the SCN and other brain regions at the molecular level.^10^ These genes include brain and muscle arnt‐like protein‐1 (*Bmal1*), circadian locomotor output cycles kaput (Clock), period circadian regulator (Per), cryptochrome circadian regulator (Cry), reverse erythroblastosis virus (Rev‐erb) and retinoic acid receptor‐related orphan receptors (Ror) for forming an auto‐regulatory feedback loop.^11^ The disrupted expression of circadian clock genes and the degeneration of the critical neurons are attributed to sleep disorders in AD.^12^ Unfortunately, there is still a lack of effective drugs to control sleep disorders in AD.^13^ It has been reported that physical activity, especially aerobic exercise, can improve cognitive function.^14^ However, it is not known whether exercise can improve sleep disturbances in AD; and it is not clear for its underlying mechanisms.

In this study, we adopted the 6‐month‐old Amyloid Precursor Protein Swedish mutation/Presenilin 1 exon 9 deletion (APP^SWE^/PS1^dE9^) transgene (TG) mouse model of AD for a long‐term voluntary wheel running (VWR) exercise for 2 months. After the VWR exercise, we found that the light‐phase hyperactivity and sleep disturbances in the AD mice were alleviated. Meanwhile, the expression of circadian clock genes in the SCN was changed. Furthermore, VWR exercise reduced phosphorylated tau (p‐tau 231) and the degeneration of the GABAergic neurons in the SCN, and upregulated the levels of beta‐site amyloid precursor protein cleaving enzyme1 (BACE1) and glycogen synthase kinase‐3β (GSK3β) in the hypothalamus. Finally, VWR exercise improved cognitive impairment and relieved the pathologic tau, Aβ accumulation, and neuroinflammation in the hippocampus and cortex of AD mice.

## 2 MATERIALS AND METHODS

### 2.1 Animals

APP^SWE^/PS1^dE9^ (TG) mice were obtained from Jackson Laboratory (Bar Harbor, United States) [B6; C3‐Tg (APPswe, PSEN1dE9) 85Dbo/Mmjax, No:004462]. The TG mice and their littermate wild‐type (WT) mice (6‐month‐old) were used in this study. The male mice were used in this study and randomly divided into four groups based on their genotypes: WT mice (WT‐Ctrl), WT mice with VWR exercise (WT‐Ex), TG mice (TG‐Ctrl), and TG mice with VWR exercise (TG‐Ex). The mice from four groups were randomly sacrificed at Zeitgeber Time (ZT)1 (9:00) and ZT13 (21:00). The number of animals used in this study is given in each method section.

Male TG mice were hybridized with female WT mice to generate offspring. At 21 days, the genotype of mice was identified by polymerase chain reaction (PCR) assay through tail biopsies. The sequence of the transgene forward primer is 5′AGGACTGACCACTCGACCAG 3′ and the transgene reverse primer is 5′CGGGGGTCTAGTTCTGCAT 3′.

All animals were housed under specific pathogen‐free conditions (temperature, 22 ± 2°C; humidity, 50% ± 10%; air exchange per 20 min; 12 h/12 h light/dark cycle with lights at 8 a.m.). All animal experiments were performed according to the guidelines approved by the Institutional Animal Care Committee of Dalian Medical University.

### 2.2 Voluntary wheel running exercise

Animals in WT‐Ex and TG‐Ex groups were housed in a cage with a running wheel (activity wheels, Shanghai Xinruan Information Technology Co, Ltd, China). Each mouse was free to access the running wheel, food, and water. To minimize the stress and depression‐like symptoms by the influence of single housing,^15^ two mice were housed in one cage. Mice in the WT‐Ctrl and TG‐Ctrl groups were housed in the same cages and conditions without the running wheel. After 2 months, the mice were housed in a single cage to record the circadian behavior and running distance for 3 days.

### 2.3 Behavioral analysis

After 2 months of VWR exercise, all four groups of mice (WT‐Ctrl, WT‐Ex, TG‐Ctrl, and TG‐Ex) were examined for cognitive function by the Morris water maze test and Y‐maze test. The time for conducting behavioral tests was consistent, at 2 p.m. in the afternoon. We analyzed the escape latency, the number of crossing target quadrants, and the percentage of total time spent in the target quadrant in the Morris water maze. We also analyzed the alternation rate in the Y‐maze based on our previous studies, as described.^16^ There were 7–11 mice in each group.

**RESEARCH IN CONTEXT**

1. **Systematic review**: We performed a systematic literature search using PubMed, focusing on the effects of exercise on Alzheimer's disease (AD)–related sleep disorders and circadian rhythm disturbances. We found a lack of reports on this topic.
2. **Interpretation**: Our findings may lead to an integrated hypothesis that exercise can regulate the circadian clock gene expression, reduce γ‐aminobutyric acid (GABA)ergic neuron degeneration in the suprachiasmatic nucleus (SCN), and improve cognitive deficits and neuropathology in AD.
3. **Future directions**: Future studies are needed to explore circadian molecule expression at different time points during the sleep–wake cycle, and determine the SCN circadian network and sleep‐related neurotransmitter changes after chronic exercise.

### 2.4 Electroencephalography/electromyography (EEG/EMG) examination and analysis

All four groups of mice (WT‐Ctrl, WT‐Ex, TG‐Ctrl, and TG‐Ex) were arranged for EEG/EMG analysis after the electrodes were implanted. Based on our previous study,^17^ we analyzed the EEG signals including the time of wake stages, rapid eye movement (REM) sleep, non‐REM (NREM) sleep,^18^ and the sleep or wake phase shifts in a 24‐h light and dark cycle. There were 3–4 mice in each group.

### 2.5 Immunofluorescence staining

After 2 months of VWR exercise, we conducted EEG/EMG examination and analysis. Then, all four groups of mice were anesthetized at 8:00–9:00 a.m. with isoflurane and perfused with pre‐cooled phosphate buffered saline (PBS, 0.1 M, pH 7.2). The brains were fixed in 4% paraformaldehyde for 24 h, followed by a dehydration process. After dehydration, the tissues were coated by Optimal Cutting Temperature Compound (Tissue‐Tek, 4583, SAKURA, Torrance, CA, USA) and sliced with the cryostat (CM‐1950S, Leica, Germany). A series of 40‐µm slices was cut coronally from the olfactory bulb to the brainstem. We used a coordination map to localize SCN, hippocampus, and cortex. The sections of SCN, hippocampus, and cortex were washed three times with PBS and incubated with blocking buffer for 1 h at room temperature, and then incubated with the primary antibodies overnight at 4°C, washed with PBS, and incubated with secondary antibodies. Finally, the sections were attached to glass slides for imaging.

We further assessed the immunofluorescence (IF) staining to detect the levels of several core ciradian proteins such as BMAL1, CLOCK, REV‐ERBα, and RORα in the SCN. Phospho‐tau protein was stained with the p‐tau231 antibody, and GABAergic neurons were stained with the vesicular GABA transporter (VGAT) antibody. The neurofilament protein heavy chain (NF‐H) was detected with the NF‐H antibody. To assess neuroinflammation in the hippocampus and cortex, microglia were stained with ionized calcium‐binding adapter molecule 1 (Iba1) antibody, and astrocytes were stained with glial fibrillary acidic protein (GFAP) antibody. IF was used to stain the Aβ plaques by 6E10 antibody. The primary antibodies are listed in Table S1. Finally, the sections were imaged using a laser scanning confocal microscope (A1confocal, Nikon, Japan) under 60× objective lenses. Data were collected from 7–11 sections per animal and 3 mice per group. The analysis of CLOCK, BMAL1, REV‐ERBα, RORα, and p‐tau231 was used to calculate the integrated density (normalized to WT‐Ctrl) by Image J software (Rockville, United States). The analysis of VGAT, NF‐H, Iba1, GFAP and 6E10 was used to calculate the area fraction (normalized to WT‐Ctrl) by Image J software. The details of IF used for this study were referred to in our previous study.^19^

### 2.6 Real‐time quantitative polymerase chain reaction (RT‐qPCR)

We then extracted the hypothalamus from four groups of mice, measured the messenger RNA (mRNA) levels of *Clock*, *Bmal1*, *Revα*, *Revβ*, *Rorα*, *Rorβ*, *Cry1*, and *Per1* by RT‐qPCR at ZT1 (9:00) and ZT13 (21:00). The tissue samples were mixed with Trizol and chloroform separately. The mixtures were grinded and centrifuged for 15 minutes. Then the supernatant was removed and mixed with isoproanol for washing. Centerfuged again, the supernatant was discarded and the remain was washed by 75% ethanol twice. After dried, the total RNA was extracted. Then we synthesized total RNA into complementary DNA (cDNA) and performed qPCR following the instructions of Hifair III 1st Strand cDNA Synthesis SuperMix (YEASEN, 11141es60, China) and Taq SYBR Green qPCR Premix (YUGONG, EG20117M, China). The sequence of primers is listed in Table S2. The relative expression levels were calculated using the 2‐ΔΔCt method. There were 3–4 mice in each group.

### 2.7 Western blotting

We performed western blotting to determine the protein levels of CLOCK and BMAL1, APP, p‐tau231, BACE1 and GSK3β, in the hypothalamus extracts obtained from four groups of mice (WT‐Ctrl, WT‐Ex, TG‐Ctrl, and TG‐Ex). The primary antibodies are listed in Table S1. The results were obtained from five mice for each group, and the experiments were repeated three times. The details of western blotting methods used for this study were referred on our previous study.^19^

### 2.8 Enzyme‑linked immunosorbent assay (ELISA)

The hypothalamus tissue from four groups of mice was dissected rapidly on ice at ZT1 (9:00) and sonicated in lysis buffer (containing 50 mM Tris pH 7.4, 150 mM sodium chloride, 1% triton X‐100, and 1% sodium deoxycholate), after grinding and centrifugation, we collected the supernatant to determine human Aβ42 and Aβ40 levels by Human Aβ42 ultrasensitive ELISA Kit (KHB3544, Thermo Fisher Scientific, Waltham, MA, USA) and Human Aβ40 ELISA Kit (KHB3481, Thermo Fisher Scientific, Waltham, MA, USA). There were four mice in each group.

### 2.9 Statistical analysis

All data were assessed using GraphPad Prism 9.0 software (GraphPad Software Inc, USA), and statistical data were expressed as means ± standard error of the mean (SEM). The data from behavioral analyses, EEG, and biochemical results were analyzed using two‐way analysis of variance (ANOVA) followed by Sidak's multiple comparisons tests. *p *< 0.05 was considered statistically significant. All experiments were repeated at least three times.

## 3 RESULT

### 3.1 VWR exercise reduces light‐phase hyperactivity and alters the sleep structure in APP^SWE^/PS1^dE9^ mice

**FIGURE 1:**
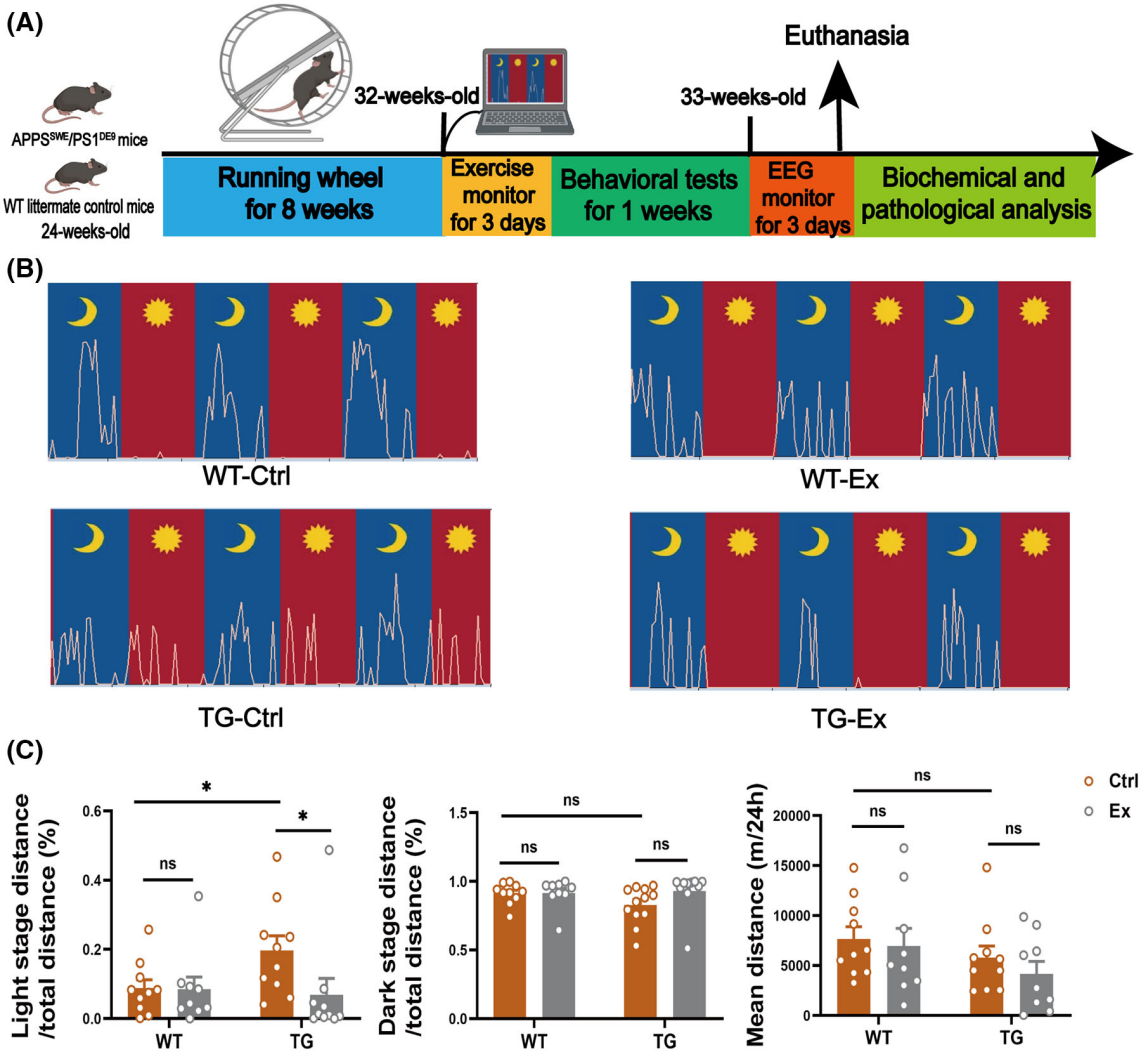
VWR exercise reduces light‐phase hyperactivity in APP^SWE^/PS1^dE9^ mice. (A) Experimental scheme for VWR exercise. (B) Representative images of behavioral circadian rhythm during 72 h for four groups of mice. The yellow curve represents the activities of one mouse. The symbol of the moon in blue represents the 12‐h dark stage, and the sun in red represents the 12‐h light stage. (C) Percentage of running distance in light or dark stages compared to total distance in 72h separately, and total distance (meter) compared to per 24h as mean distance per 24 h. *n* = 7–11 mice in each group. Data were analyzed by two‐way ANOVA and presented as the mean ± SEM. ns, non‐significant, *p < 0.05. ANOVA, analysis of variance; EEG, electroencephalogram.

TG‐Ctrl mice showed higher activities than the WT‐Ctrl mice during the 12‐h light stage (Figure 1B); the running distance was significantly increased by 123% in TG‐Ctrl mice (*p *< 0.05; Figure 1C). After 2 months of VWR exercise, the running distance was markedly decreased by 64.9% in the 12‐h light stage in the TG‐Ex mice versus the TG‐Ctrl mice (*p *< 0.05; Figure 1C). There was no significant difference between the four groups in the running distance during the 12‐h dark stage and the mean distance per day. As mice primarily have nocturnal activity and sleep during the daytime, the VWR exercise reduced diurnal activity and improved the behavioral circadian rhythm disorder in the TG‐Ex mice.

**FIGURE 2:**
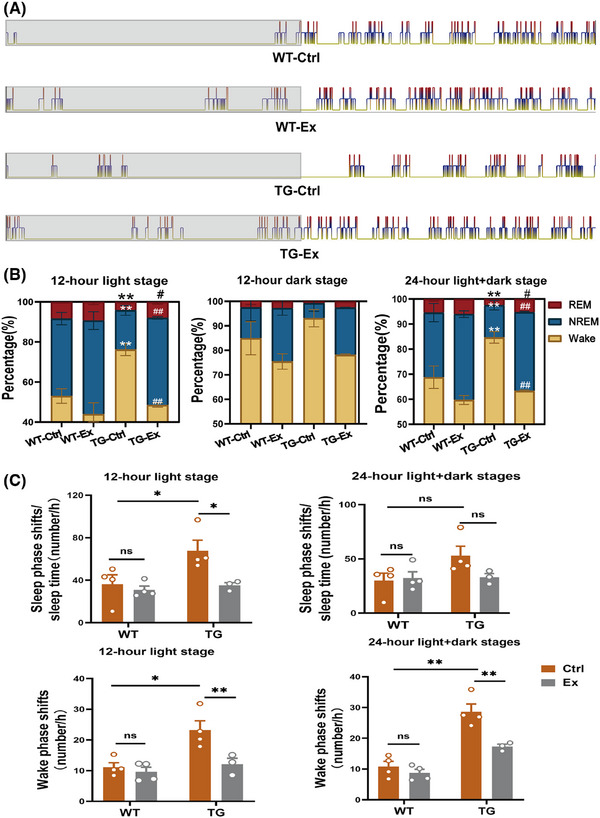
VWR exercise alters the sleep structure in APP^SWE^/PS1^dE9^ mice. (A) Representative images of different sleep phages during 24‐h light and dark stages. The gray background represents the 12‐h dark stage and the white background represents the 12‐h light stage. The red line represents REM sleep, the blue line represents NREM sleep, and the yellow line represents the wake stages. (B) Quantification of the percentage time of REM, NREM, and wake stages compared with the 12‐h light stage, 12‐h dark stage, and 24‐h light and dark stages, respectively. *: compared with WT‐Ctrl group; #: compared with TG‐Ctrl group. *p < 0.05, **p < 0.01, ^#^ *p *< 0.05, ^##^ *p *< 0.01. (C) Quantification of the sleep phase shifts (NREM to REM, REM to NREM)/sleep time, wake phase shifts (NREM to wake, REM to wake)/h in the 12‐h light stage and 24‐h light and dark stages, respectively. *n* = 3–4 mice per group. Data were analyzed by two‐way ANOVA and presented as the mean ± SEM. ns, non‐significant, *p < 0.05, **p < 0.01. NREM sleep, non‐rapid eye movement sleep; REM sleep, rapid eye movement sleep.

To further investigate sleep structure change we recorded day–night EEG in all four groups of mice. We found that the percentage of REM sleep in the TG‐Ctrl mice was significantly decreased by 50.6%; the NREM sleep was decreased by 49.2%; and wakefulness was increased by 43.2% during the 12‐h light stage versus the WT‐Ctrl mice (both *p* < 0.01; Figure 2B). After VWR exercise, the percentage of REM sleep was significantly increased by 89% (*p *< 0.05), the NREM sleep was increased by 119%, and the wake stages were decreased by 36.8% during the 12‐hour light stage in the TG‐Ex mice versus the TG‐Ctrl mice (both *p *< 0.01; Figure 2B).

Furthermore, we documented that the number of sleep phase shifts (NREM to REM, REM to NREM) was increased by 109%, and wake phase shifts (NREM to wake, REM to wake) was increased by 107% during the 12‐h light stage in the TG‐Ctrl mice versus the WT‐Ctrl mice (both *p *< 0.05; Figure 2C). After the VWR exercise, the sleep phase shifts were decreased by 47.7% (*p *< 0.05; Figure 2C), and the wake phase shifts were decreased by 47.1% (*p *< 0.01; Figure 2C) during the 12‐h light stage in the TG‐Ex mice versus the TG‐Ctrl mice. These findings suggest that VWR exercise improved the disturbance in sleep structure and decreased sleep fragmentation, especially during the light stage.

### 3.2 VWR exercise improves cognitive impairment in APP^SWE^/PS1^dE9^ mice

**FIGURE 3:**
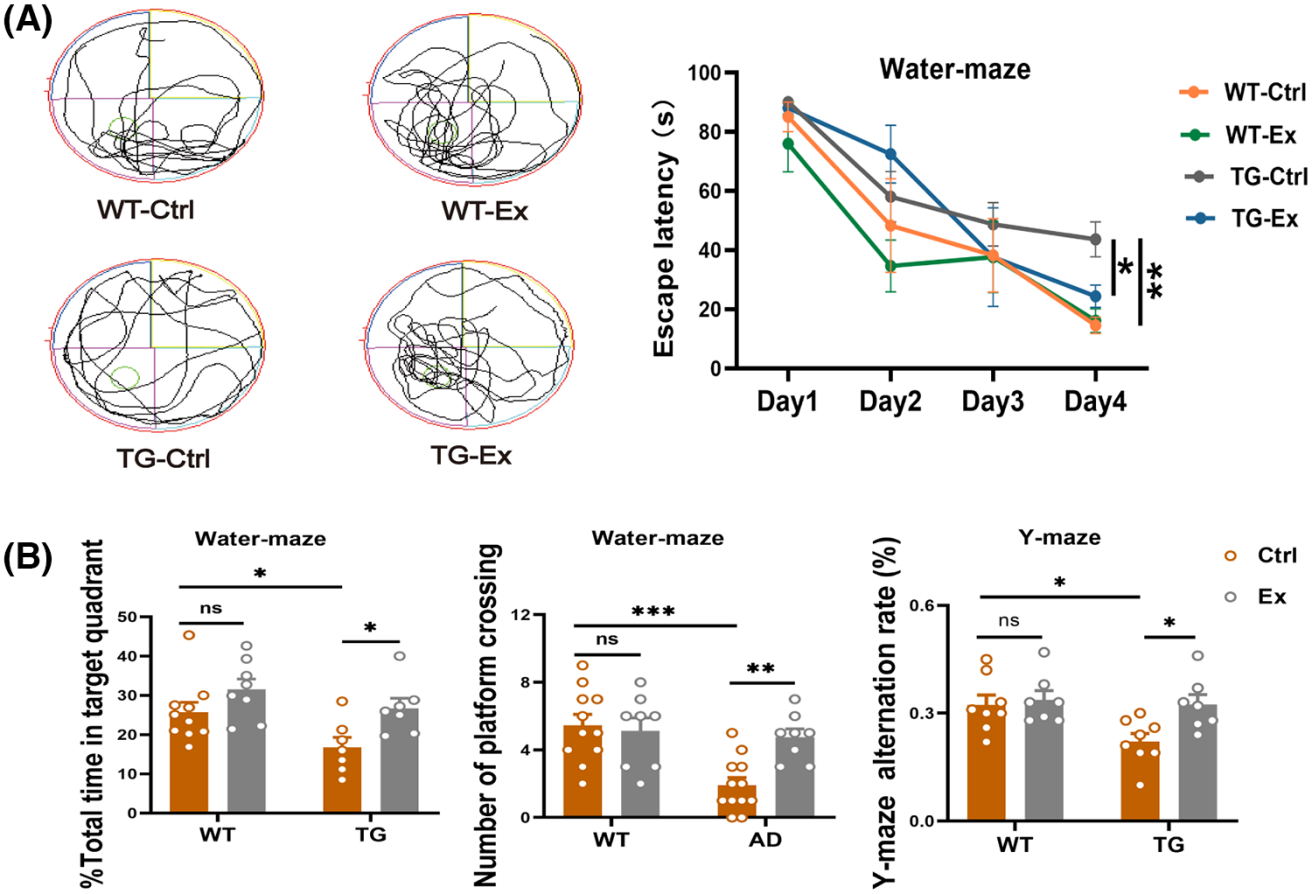
VWR exercise improves cognitive impairment in APP^SWE^/PS1^dE9^ mice. (A) Representative images of Morris water maze for four groups of mice and the escape latency. (B) The number of crossings in the target quadrant and the percentage of total time spent in the target quadrant in Morris water maze. Percentage of alternation rate in the Y‐maze experiment. *n* = 7–11 mice per group. Data were analyzed by two‐way ANOVA and presented as the mean ± SEM. ns, non‐significant, *p < 0.05, **p < 0.01, ***p < 0.001.

To evaluate the effect of VWR exercise on the cognitive function in the TG mice, we conducted the Morris water maze and Y‐maze tests. They showed that TG‐Ex mice displayed a significant decrease in escape latency on day 4 (by 44%, *p *< 0.05; Figure 3A), a significant increase in the number of platform crossings (by 147%, *p *< 0.01; Figure 3A‐B), and total time in the target quadrant (by 59%, *p *< 0.05; Figure 3A‐B) compared with TG‐Ctrl mice. Consistently, the Y‐maze alternation percentage was increased markedly in the TG‐Ex mice by 46.5% versus the TG‐Ctrl mice (*p *< 0.05; Figure 3B). There was no significant difference between the WT‐Ctrl and the WT‐Ex mice in the Morris water maze and Y‐maze tests. Together, our results reveal that VWR exercise can improve the learning and memory abilities in the TG mice.

### 3.3 VWR exercise changes the expression of circadian clock genes in the SCN of APP^SWE^/PS1^dE9^ mice

**FIGURE 4:**
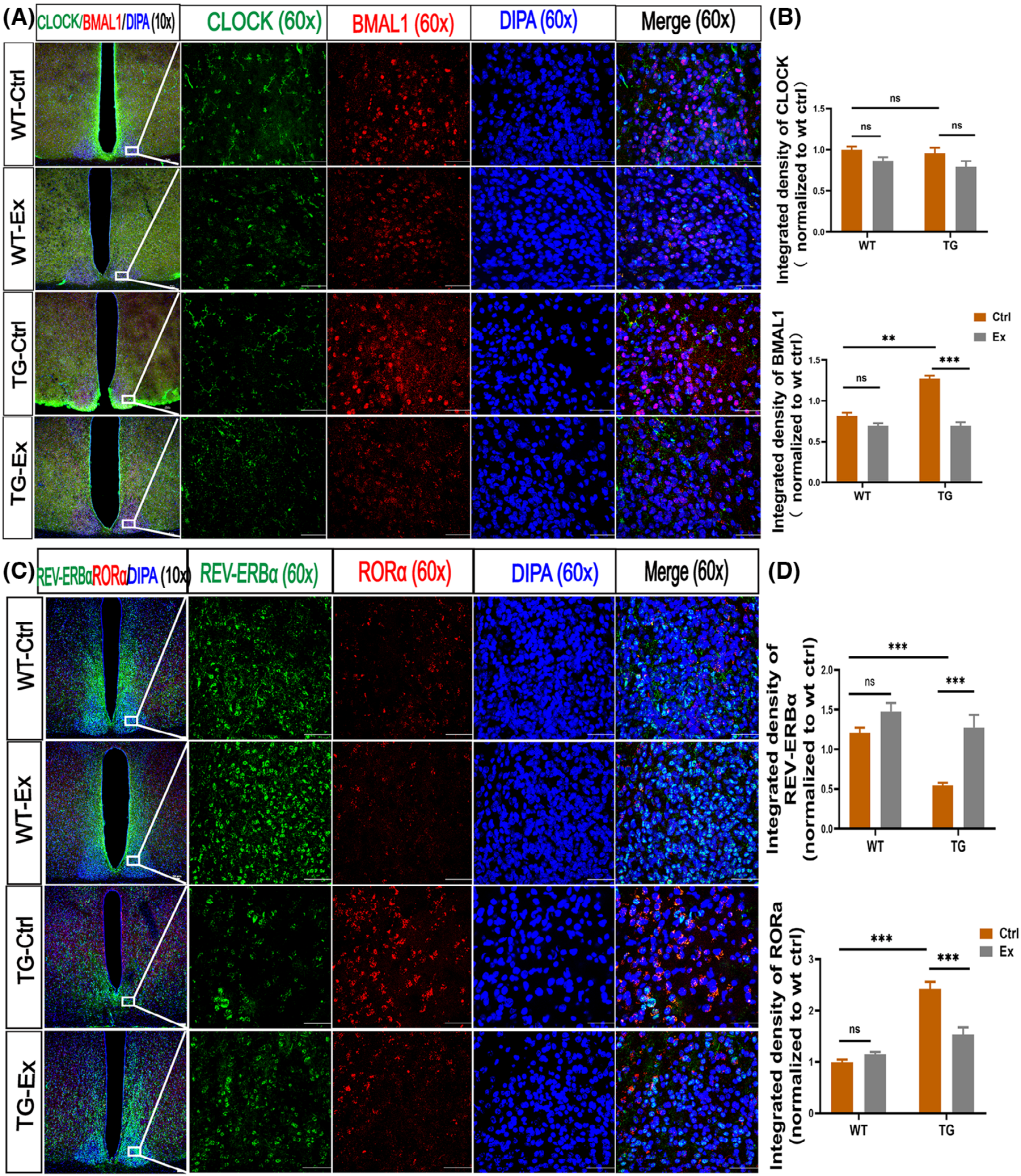
VWR exercise changes the expression of circadian clock genes in the SCN of APP^SWE^/PS1^dE9^ mice. (A) Immunofluorescence analysis for CLOCK expression (green) in the SCN together with BMAL1 (red). The nucleus was labeled with DAPI (blue). (B) Quantification of the integrated density (normalized to WT‐Ctrl) of CLOCK, BMAL1. (C) Immunofluorescence analysis for REV‐ERBα expression (green) in the SCN together with RORα (red). The nucleus was labeled with DAPI (blue). (D) Quantification of the integrated density (normalized to WT‐Ctrl) of REV‐ERBα, RORα. *n* = 3 mice per group, scale bars 50 um. Data were analyzed by two‐way ANOVA and presented as the mean ± SEM. ns, non‐significant, **p < 0.01, ***p < 0.001. BMAL1, brain and muscle arnt‐like protein‐1; CLOCK, circadian locomotor output cycles kaput; DAPI, 4'6‐diamidino‐2‐phenylindole; REV‐ERBα, reverse erythroblastosis virusα; RORα, retinoic acid receptor‐related orphan receptorsα; SCN, suprachiasmatic nucleus.

SCN in the hypothalamus is the master circadian pacemaker that maintains circadian outputs. We focused on the core circadian clock genes in the SCN and observed an increased expression of BMAL1 by 46.9% (*p *< 0.01; Figure 4A‐B) in the TG‐Ctrl mice versus the WT‐Ctrl mice. After the VWR exercise, the expression of BMAL1 was decreased by 45.7% in the TG‐Ex mice versus the TG‐Ctrl mice (*p *< 0.001; Figure 4A‐B). Furthermore, we measured the expression of CLOCK in the SCN of four groups and found no significant difference.

In addition, we measured the expressions of REV‐ERBα and RORα, which regulate the BMAL1. We found a 54.7% reduction in the expression of REV‐ERBα in the TG‐Ctrl mice versus the WT mice in the SCN (*p *< 0.001; Figure 4C–D). After VWR exercise, the expression of REV‐ERBα was increased by 119% in the TG‐Ex mice versus the TG‐Ctrl mice (*p *< 0.001; Figure 4C‐D). Meanwhile, the level of RORα was significantly increased by 116% in the TG‐Ctrl mice versus the WT‐Ctrl mice. After VWR exercise, it was decreased by 36.4% in the TG‐Ex mice versus the WT‐Ex mice (both *p* < 0.001; Figure 4C‐D). These results indicate that VWR exercise can change the expression levels of the core circadian clock genes.

### 3.4 VWR exercise decreases the tau phosphorylation and GABAergic neuron degeneration in the SCN of APP^SWE^/PS1^dE9^ mice

**FIGURE 5:**
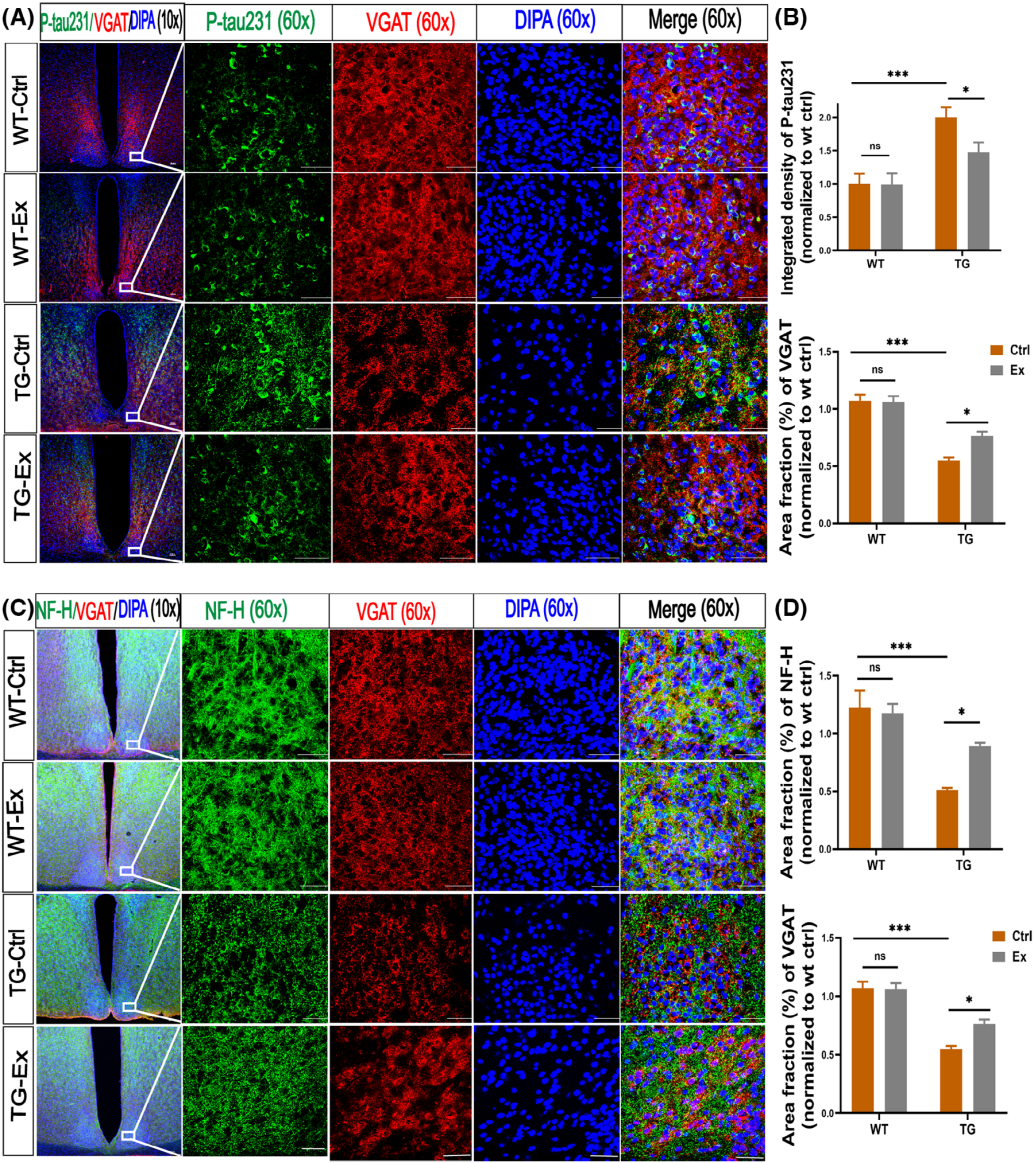
VWR exercise decreases the tau phosphorylation and GABAergic neuron degeneration in the SCN of APP^SWE^/PS1^dE9^ mice. (A) Immunofluorescence analysis for p‐tau231 expression (green) in the SCN together with VGAT (red). The nucleus was labeled with DAPI (blue). (B) Quantification of the integrated density (normalized to WT‐Ctrl) of p‐tau231, area fraction (%) (normalized to WT‐Ctrl) of VGAT. (C) Immunofluorescence analysis for NF‐H expression (green) in the SCN together with VGAT (red). The nucleus was labeled with DAPI (blue). (D) Quantification of the area fraction (%) (normalized to WT‐Ctrl) of NF‐H, VGAT. *n* = 3 mice per group, scale bars 50 um. Data were analyzed by two‐way ANOVA and presented as the mean ± SEM. ns, non‐significant, **p < 0.01, ***p < 0.001. NF‐H, neurofilament protein heavy chain; p‐tau231, phosphor‐tau; VGAT: vesicular GABA transporter.

Hyperphosphorylated tau protein aggregated in the cytoplasm is a typical hallmark of AD. Because most neurons in the SCN are GABAergic,^20^ we further elucidate the pathological changes in the SCN. A significant increase in the integrated density of intracellular p‐tau231 was found in the SCN of TG‐Ctrl mice versus WT‐Ctrl mice (by 129%, *p *< 0.001; Figure 5A,B). Meanwhile, the VGAT, a marker of GABAergic neurons, was decreased by 94.5% in the TG‐Ctrl mice versus the WT‐Ctrl mice (*p *< 0.001; Figure 4A,B). Double‐staining showed that some p‐tau231 in the cytoplasm was co‐localized with GABAergic neurons in the TG mice (Figure 5A). After VWR exercise, the integrated density of p‐tau231 was markedly reduced by 35% in the TG‐Ex mice versus the TG‐Ctrl mice (*p *< 0.05; Figure 5A,B). It is important to note that the VGAT was increased by 38.7% in the TG‐Ex mice versus the TG‐Ctrl mice (*p *< 0.05; Figure 5A,B).

NF‐H is one of the important components of cytoskeletal proteins in the large myelinated axons, which is often released into the extracellular fluid due to axonal damage in AD.^21^ We found that the integrated density of NF‐H was reduced by 140% in the SCN of TG‐Ctrl mice versus WT‐Ctrl (*p *< 0.001; Figure 5C,D). After VWR exercise, the integrated density was elevated by 74.5% in the TG‐Ex mice versus the TG‐Ctrl mice (*p *< 0.05; Figure 5C,D). Furthermore, NF‐H was co‐localized with VGAT in the SCN, indicating that VWR exercise reduces the degeneration of GABAergic neurons in the TG mice.

### 3.5 VWR exercise alters the expression of circadian clock genes and reduces APP protein and tau protein phosphorylation in the hypothalamus of APP^SWE^/PS1^dE9^ mice

**FIGURE 6:**
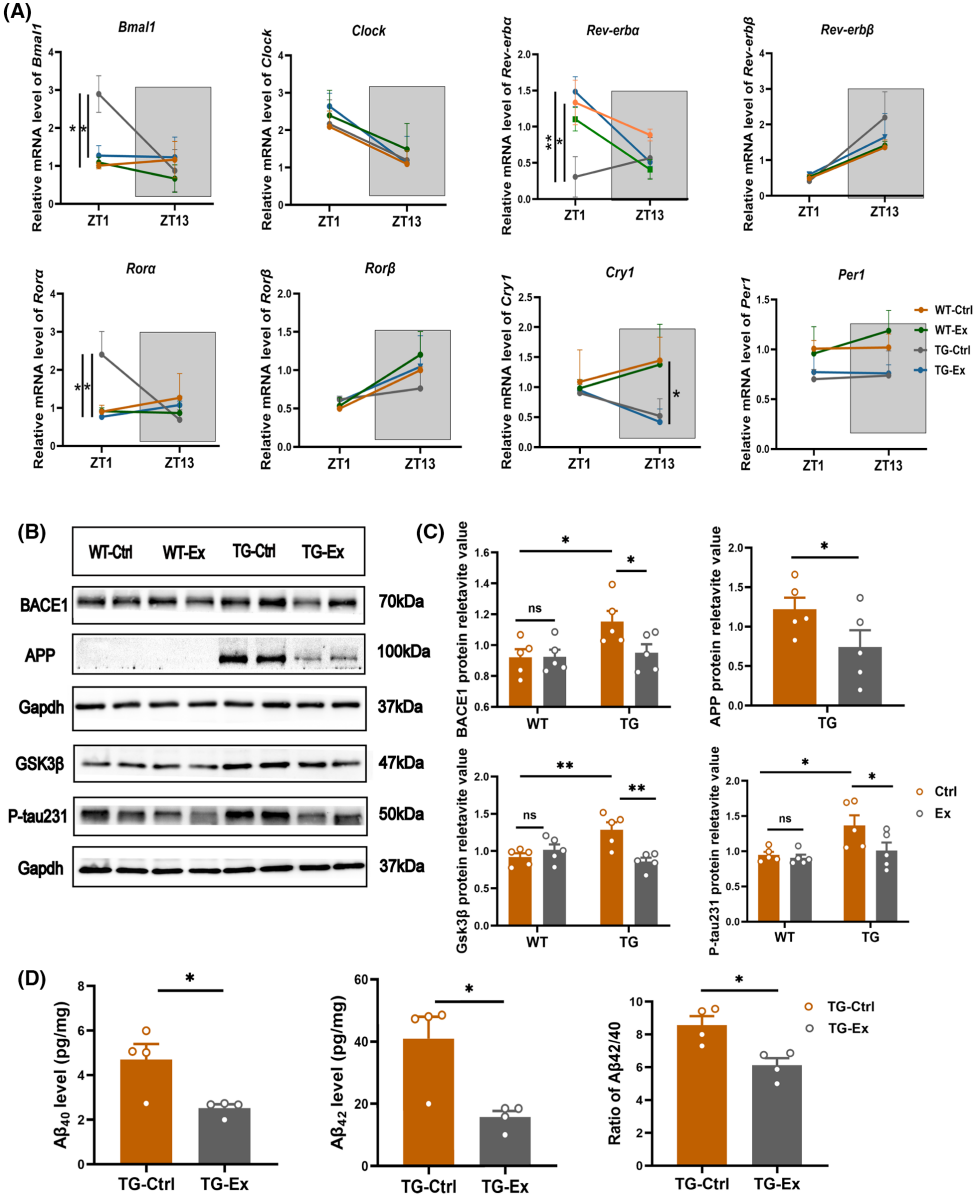
VWR exercise alters the expression of circadian clock genes, reduces APP protein, and tau protein phosphorylation in the hypothalamus of APP^SWE^/PS1^dE9^ mice. (A) The mRNA levels of clock genes (*Bmal1*, *Clock*, *Rev‐erbα*, *Rev‐erbβ*, *Ror*α, *Ror*β, *Cry1*, *Per1*) in the hypothalamus. The white background represents the light stage, whereas the gray background represents the dark stage. *n* = 3–4 mice per group. (B) Western blot analysis for BACE1 (BACE1 ab), APP (6E10 ab), GSK3β (GSK3β ab), p‐tau231 (phospho‐tau231 ab), and Gapdh (GAPDH ab) in the hypothalamus. *n* = 5 mice per group. (C) Quantification of BACE1, APP, GSK3β, and p‐tau231 expression relative to Gapdh. Multiple group comparisons were analyzed by two‐way ANOVA. (D) Quantification of Aβ40, Aβ42 levels, and ratio of Aβ42/40 by ELISA in the hypothalamus. *n* = 4 mice per group. Two‐group comparisons were analyzed by Student's *t*‐test. Experiments were repeated three times. Data are presented as the mean ± SEM. ns, non‐significant, *p < 0.05, **p < 0.01, ***p < 0.001. ab, antibody; Aβ40, amyloid‐β40; Aβ42, amyloid‐β42; APP, amyloid precursor protein; BACE1, beta‐site amyloid precursor protein cleaving enzyme 1; Cry, cryptochrome circadian regulator; ELISA, enzme‐linked immunsorbent assay; GAPDH: glyceraldehyde‐3‐phosphate dehydrogenase; GSK3β, glycogen synthase kinase‐3β; Per, period circadian regulator.

Because the expression of core clock genes changed in SCN, we further examined the mRNA level of clock genes in the hypothalamus at two time points: ZT1 (9:00) and ZT13 (21:00). We found that the abnormal expression of clock genes mainly occurred during the light stage in the TG mice, and VWR exercise significantly alleviated such changes (Figure 6A).

At ZT1, we found an increased expression of *Bmal1* and *Ror*α (by 180% and 169%, both *p *< 0.05; Figure 6A) in the TG‐Ctrl mice versus the WT‐Ctrl mice. After the VWR exercise, the expression decreased by 57% and 68% in the TG‐Ex mice versus the TG‐Ctrl mice (both *p *< 0.05; Figure 6A). In addition, the expressions of *Rev‐erbα* were reduced by 77% (*p *< 0.05) in the TG‐Ctrl mice versus the WT mice, and elevated by 79% after VWR exercise in the TG‐Ex mice versus the TG‐Ctrl mice (*p *< 0.01; Figure 6A). There were no significant changes in the expression of other clock genes (*Clock*, *Rev‐erbβ*, *Rorβ*, *Cry1*, *Per1*). This tendency in the hypothalamus was similar to that in the SCN. At ZT13, only the mRNA level of *Cry1* declined by 65% in the TG‐Ctrl mice versus the WT‐Ctrl mice (*p *< 0.05; Figure 6A), and there was no significant change after VWR exercise (Figure 6A).

Furthermore, we found that the expression of p‐tau231 in the hypothalamus was reduced by 25.7% (*p *< 0.05; Figure 6B‐C), and the level of APP protein was reduced by 32.3% (*p *< 0.01; Figure 6B‐C) in the TG‐Ex mice versus the TG‐Ctrl mice. In addition, we measured the level of Aβ40 and Aβ42 in the hypothalamus by ELISA. We found a significant reduction of Aβ40 (by46.8%), Aβ42 (by 62.5%), and Aβ42/Aβ40 ratio (by 28.8%) in the TG‐Ex mice versus the TG‐Ctrl mice (all of them *p *< 0.05; Figure 6D).

BACE1 is a rate‐limited enzyme for Aβ production and plays a critical role in AD pathology.^22^ Meanwhile, we found that the expression of BACE1 was decreased by 26.7% in the TG‐Ex mice versus the TG‐Ctrl mice (*p *< 0.05; Figure 6B‐C). The GSK3β promotes tau hyperphosphorylation and induces Aβ formation.^23^ We also found that the expression of GSK3β was lower by 26.5% in the TG‐Ex mice compared to the TG‐Ctrl mice (*p *< 0.01; Figure 6B‐C), suggesting that VWR exercise may reduce the pathological accumulation via suppression of BACE1 and GSK3β in the hypothalamus of TG mice.

### 3.6 VWR exercise reduces the tau phosphorylation in the hippocampus and cortex of APP^SWE^/PS1^dE9^ mice

**FIGURE 7:**
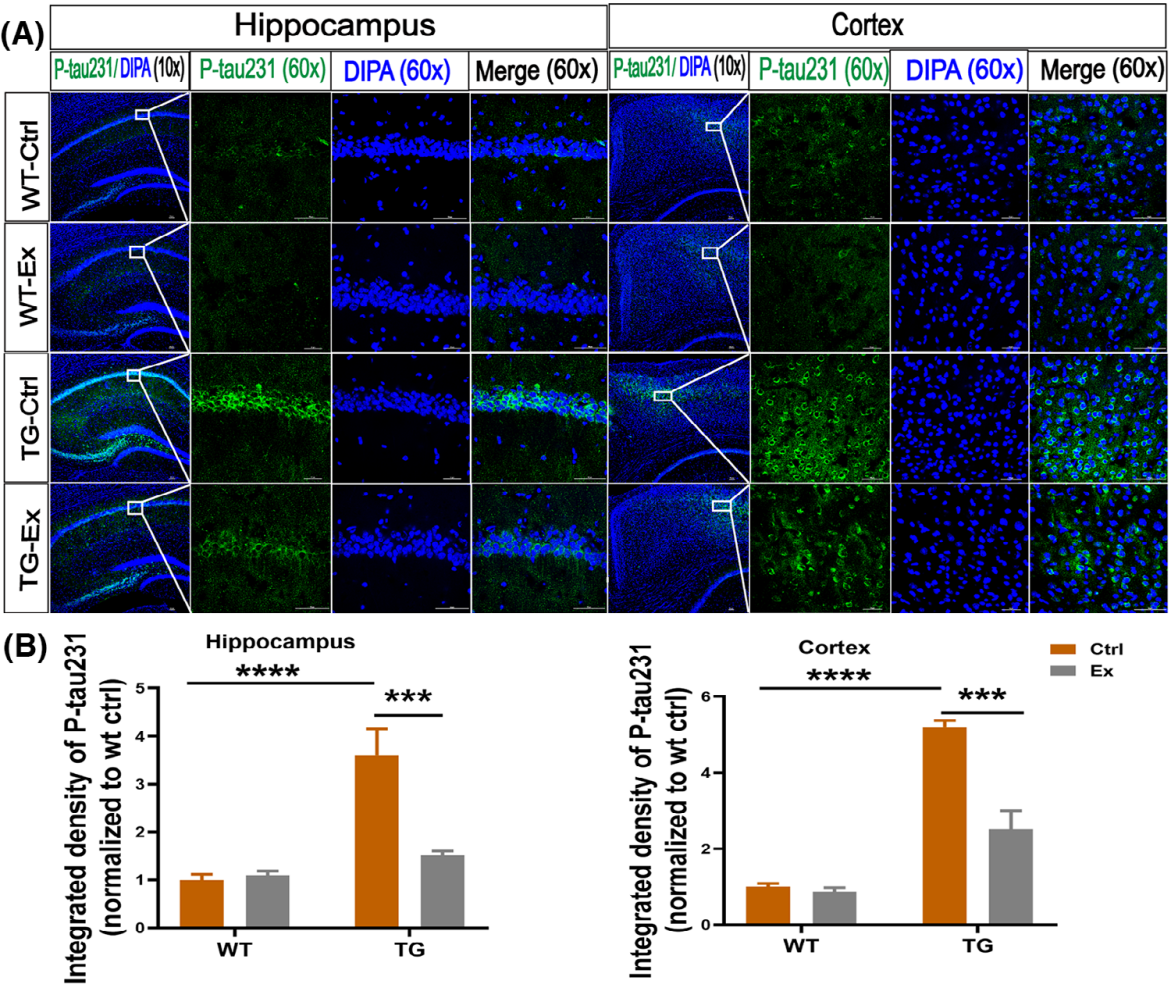
VWR exercise reduces the tau phosphorylation in the hippocampus and cortex of APP^SWE^/PS1^dE9^ mice. (A) Immunofluorescence analysis for p‐tau231 expression (green) in the hippocampus and cortex. The nucleus was labeled with DAPI (blue). (B) Quantification of integrated density (normalized to WT‐Ctrl) of p‐tau231 in the hippocampus and cortex. *n* = 7–11 slices per mouse and 3 mice per group, scale bars 50 um. Data were analyzed by two‐way ANOVA and presented as the mean ± SEM. ns, non‐significant, *p < 0.05, **p < 0.01, ***p < 0.001, ****p < 0.0001.

Next, we measured the p‐tau231 protein by IF staining and found that the integrated density of p‐tau231 was increased significantly in the hippocampus (by 259%, *p *< 0.0001) and cortex (by 419%, *p *< 0.0001) in the TG‐Ctrl mice versus WT‐Ctrl mice (Figure 7A‐B). In addition, we documented a marked decrease of p‐tau231 IF staining after VWR exercise in the hippocampus (by 57.1%) and cortex (by 51.8%) in the TG‐Ex mice versus the TG‐Ctrl mice (both *p *< 0.001; Figure 7A‐B). These results were consistent with the change of p‐tau231 in the SCN.

### 3.7 VWR exercise reduces neuroinflammation and Aβ plaque accumulation in the hippocampus and cortex of APP^SWE^/PS1^dE9^ mice

**FIGURE 8:**
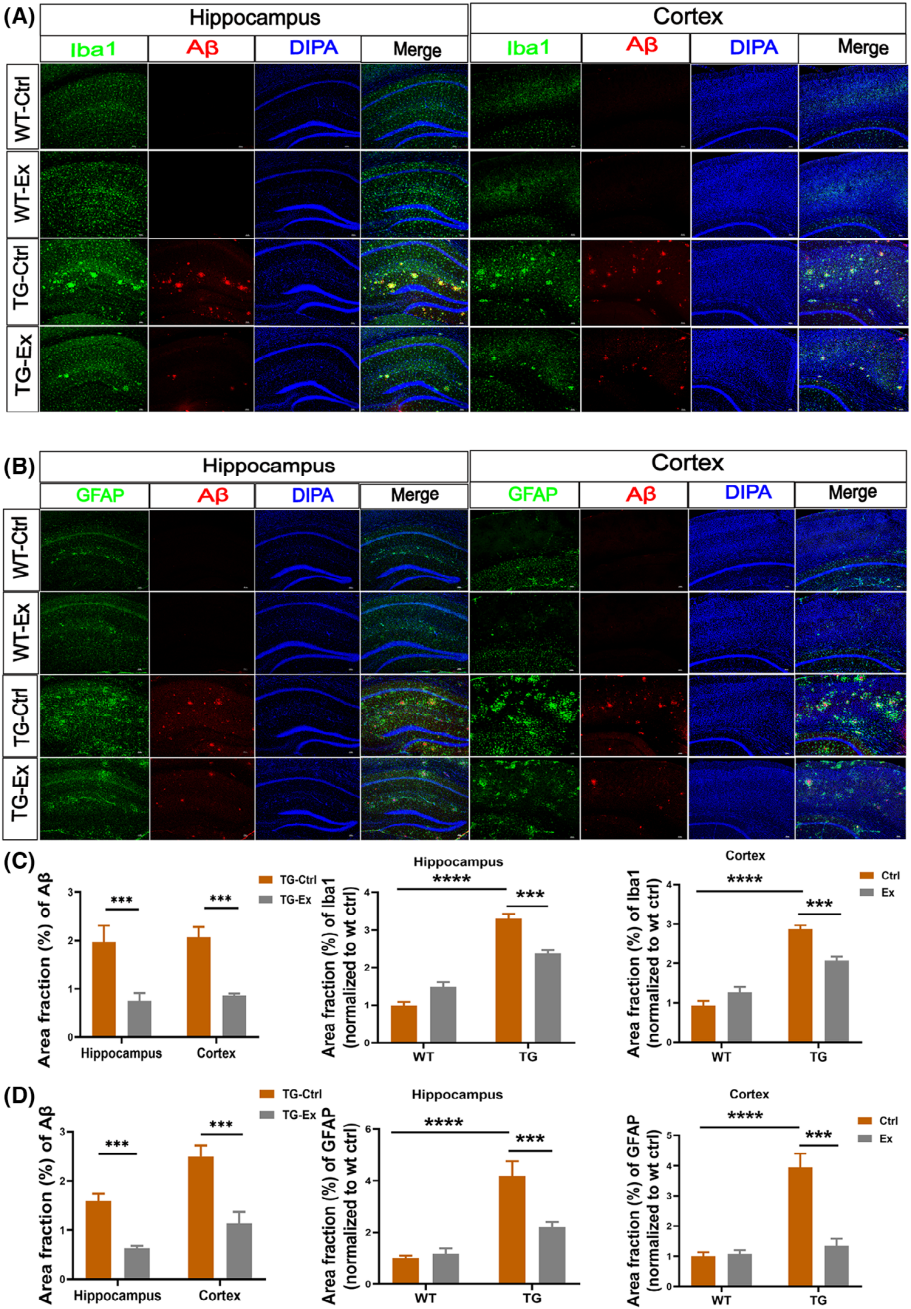
VWR exercise reduces neuroinflammation, Aβ plaque accumulation in the hippocampus and cortex of APP^SWE^/PS1^dE9^ mice. (A) Immunofluorescence analysis for Iba1 expression (green) together with Aβ (red) in the hippocampus and cortex. The nucleus was labeled with DAPI (blue). (B) Immunofluorescence analysis for GFAP expression (green) together with Aβ (red) in the hippocampus and cortex. The nucleus was labeled with DAPI (blue). (C‐D) Quantification of the area fraction (%) of Aβ, Iba1, and GFAP in the hippocampus and cortex. *n* = 7–11 slices per mouse and 3 mice per group, scale bars 50 um. Data were analyzed by two‐way ANOVA and presented as the mean ± SEM. ns, non‐significant, ***p < 0.001, ****p < 0.0001. GFAP, glial fibrillary acidic protein; Iba1, ionized calcium‐binding adapter molecule 1.

In our study, we documented that VWR exercise improved cognitive impairment in the TG mice. Then we further assessed the neuropathological changes of the hippocampus and cortex of TG mice. TG‐Ctrl mice showed abundant Aβ plaque in the hippocampus and cortex (Figure 8A‐B). After VWR exercise, it was decreased significantly in the hippocampus (by 60.4%) and cortex (by 57%) in the TG‐Ex mice versus the TG‐Ctrl mice (both *p *< 0.001, Figure 8C). It is well known that neuroinflammation plays an important role in the pathogenesis of AD.^24^ We then examined the levels of microglia with the Iba1 immunofluorescent staining and astrocytes with the GFAP immunofluorescent staining. We found that microglia and astrocytes were concentrated around the Aβ plaques. The area fraction of Iba1 was higher in the hippocampus (by 230%, *p *< 0.0001) and cortex (by 180%, *p *< 0.0001) of TG‐Ctrl mice versus WT‐Ctrl mice (Figure 8A‐C). Similarly, the area fraction of GFAP was elevated in the hippocampus (by 318%) and cortex (by 294%) in the TG‐Ctrl mice versus WT‐Ctrl mice (both *p *< 0.0001; Figure 8B‐D).

After the VWR exercise, we found that the area fraction (%) of Iba1 was markedly downregulated in the hippocampus (by 27.9%) and cortex (by 28.6%) in the TG‐Ex mice versus the TG‐Ctrl mice (both *p *< 0.001, Figure 8A‐C). In addition, the area fraction (%) of GFAP was reduced in the hippocampus (by 47.2%) and cortex (by 66.7%) in the TG‐Ex mice versus the TG‐Ctrl mice (both *p *< 0.001, Figure 8B‐D).

## 4 DISCUSSION

In this study, we first demonstrated that long‐term VWR exercise improved behavioral circadian rhythm and sleep disorders in AD mice. The results showed that the sleep–wake cycle became more regular in the TG‐Ex mice; the total awakening time and sleep fragmentation were reduced during the light stage. Furthermore, we found that VWR exercise affected the expression of core clock genes in the SCN. Meanwhile, the VWR exercise reduced tau phosphorylation and axon damage of GABAergic neurons in the SCN of TG mice. In addition, changes were found in the hypothalamus accompanied by the decreased levels of BACE1 and GSK3β. Finally, VWR exercises improved cognitive impairment, reduced tau phosphorylation, and decreased the Aβ plaque deposition combined with microglia and astrocyte infiltration in the hippocampus and cortex of TG mice.

VWR exercise is a behavioral intervention widely used for mice to mimic human physical exercise training.^25^ It has been reported that exercise is an effective therapy for sleep disorders in older people.^26^ A systematic review included 3278 persons with AD showing beneficial effects of sleep disorders and cognitive dysfunction during daily exercise.^27^ In addition, increasing evidence has demonstrated that exercise improves the cognitive decline of AD, by elevated neurotrophins levels, reduced neuroinflammation, and ameliorated autophagy and neuroplasticity.^14^ However, the effect of exercise improving sleep–wake cycle disturbances in AD remains to be further studied.

In AD patients, long nocturnal awakenings and total sleep time are reduced, and NREM sleep and REM sleep are decreased. Increased daytime activities to minimize daytime naps and exposure to light are usually beneficial for sleep disturbances in AD.^28^ Our previous study found sleep structure changes before the cognitive decline in AD mice, showing that the percentage of wake stage was increased and NREM sleep was decreased.^29^ Sleep disruption and increased wakefulness could enhance Aβ production and reduce Aβ clearance,^30^ and increasing excitatory neuronal activity significantly elevated interstitial fluid tau.^31^ Aβ levels were significantly increased during the dark period compared to the light period in AD mice.^32^ In the present study, VWR exercise might alleviate AD pathologies by modifying the sleep structure in the TG mice, such as reducing the wake stage time and increasing the percentage of NREM and REM sleep in the light stage. In addition, the number of wake phase shifts decreased, which means sleep fragmentation improvement during the daytime.

SCN is a major pacemaker of the circadian system, driving circadian rhythmicity in other brain areas and peripheral tissues.^33^ It has been reported that the functional disruption of the SCN was observed from the earliest AD stages.^34^ In AD patients, the cell number in the SCN is decreased in post‐mortem tissue examination, which is associated with sleep fragmentation measured before death.^35^ There are nearly 20,000 neurons in the SCN and almost all are GABAergic. Environmental light–dark conditions and other factors, such as exercise, may coordinate the cellular synchrony rhythms in the SCN by altering GABAergic signaling.^36^ The SCN‐specific VGAT‐depleted mice show a circadian behavioral rhythm disorder.^8^ Furthermore, tauopathy within the SCN may disrupt circadian clock gene function both at the behavioral and molecular levels.^37^ Eliminating phospho‐tau in GABAergic neurons improves behavioral rhythms and cognitive deficits.^38^ In addition, the phospho‐tau accumulation in GABAergic neurons contributes to neurogenic transmission deficits in the hippocampus.^39^ In the current study, we found that after VWR exercise, the level of p‐tau in the SCN of TG mice was decreased, and the VGAT‐labeled GABAergic neurons increased. This indicated that exercise alleviates AD‐related neurodegeneration in the SCN and prevents GABAergic neuron degeneration. By preserving SCN circadian output capacity, VWR exercise intervention could stabilize the core circadian pacemaker system. The running distance in the light phase decreased in the TG‐Ex mice, corresponding to the reduction of wake time in EEG results, providing evidence for the reset of circadian rhythm. The locomotor activity of TG‐Ex mice exhibited phase consistency with that of WT‐Ctrl mice, which may indicate the restoration of the SCN‐mediated photic entrainment pathways.

The sleep–wake cycle is regulated by a group of circadian clock genes in the SCN.^40^ During the light stage, we identified the change of expression levels ofclock genes in the SCN and hypothalamus of TG‐Ex mice. During the dark period, we found only that the level of *Cry1* mRNA in the hypothalamus of TG mice was abnormal compared to that in WT mice, and there was no significant alteration after exercise. It seems that VWR exercise regulates the expression of clock genes mainly during the light stage in TG mice. The changes in the expression levels of clock genes observed in the SCN of TG mice may suggest a disorder in molecular clock oscillation patterns. And the VWR exercise seems to reset and stabilize the circadian transcriptional–translational feedback loops (TTFLs) in the SCN, which will further reset the peripheral oscillator, thereby improving sleep–wake synchronization.

Clock genes regulate cellular antioxidant responses^41^ and neuroinflammation,^42^ and are involved in maintenance of the blood–brain barrier integrity in the brain.^43^ Emerging evidence reveals a bidirectional interplay between circadian disruption and AD pathogenesis. Although molecular clock dysregulation exacerbates AD progression, accumulating Aβ pathology and hyperphosphorylated tau can also impair circadian TTFLs, thereby forming a pathogenic cycle. In our previous study, we found that chronic sleep deprivation aggravated AD pathologies and caused abnormal expression of clock genes in the circadian rhythm–related nuclei of experimental mice, and p‐tau levels were parallel to the BMAL1 levels.^44^ The increase of Aβ affected the functioning of the endogenous clock and daily rhythms of BMAL1, RORα, and their target genes.^45^ It has been reported that Aβ uptake by BV‐2 cells varies with time of day in parallel with BMAL1 expression. At the same time, pharmacological inhibition of REV‐ERBs accelerated microglial uptake of fibrillary Aβ1‐42 and increased transcription of BMAL1.^46^ However, *Rev‐erbα* deletion caused spontaneous microglial activation in the hippocampus and secondary astrogliosis.^42^ In this study, we hypothesized that the decrease in BMAL1 levels and the increase in REV‐ERBα levels in the SCN of APP/PS1 mice after VWR exercise may be related to the reduction of Aβ levels. The reduction of Aβ levels might lead to a decrease in the demand for Aβ uptake by microglia, which may further increase REV‐ ERBα levels and inhibit the transcription of BMAL1. Exercise may play an important neuroprotective role. Exercise regulates the expression of AD‐related genes or proteins through various epigenetic modulations.^47^ In addition, exercise may increase the expression of ubiquitin carboxyl‐terminal hydrolase L1,^48^ enhance the degradation of APP and BACE1 through the ubiquitin‐proteasome pathway, and decrease the Aβ level in the hippocampus of APP^SWE^/PS1^dE9^ mice.^49^ In addition, exercise can upregulate the sirtuin‐1 signaling pathway to activate PGC‐1α expression and decrease the level of BACE1.^50^ Furthermore, exercise induces the expression of the brain‐derived neurotrophic factor, which can reduce BACE1 activity directly.^51^ In AD, phosphorylation sites of tau protein mainly concentrate on the residues of serine/threonine and proline. GSK3β promotes tau aggregation by phosphorylating the sites of tau. Exercise regulates BDNF/TrkB/Akt/GSK3β/ signaling pathways and inactivates GSK3β to reduce tau phosphorylation.^52^ VWR exercise relieves the neuropathological deposition of AD in the hypothalamus, which is the most important output terminal from the SCN neural network.^53^ Microglia exist in different phenotypes, including pro‐inflammatory (M1) and anti‐inflammatory (M2).^54^ Exercise seems to be effectively promoting a phenotype from M1 to M2 and enhancing the clearing of the Aβ plaque through histone H3 acetylation.^55^ In addition, exercise promotes microglial glucose metabolism and morphological plasticity by inhibiting TREM2 shedding.^56^ In addition, exercise may regulate the astrocyte phenotype–associated aquaporin channel 4 polarization, which is a membrane‐bound water‐channel protein located at the perivascular and determines the function of the glymphatic system. These changes promote Aβ clearance from the brain tissue through the glymphatic system.^57^

In summary, the VWR exercise improves cognitive deficits, behavioral circadian rhythm disorder, and sleep structure disturbance in the AD mice. Strikingly, the VWR exercise relieves neuroinflammation and Aβ and phospho‐tau protein accumulation in the hippocampus and cortex. In addition, VWR exercise changes the expression of clock genes, and reduces cell loss and axonal damage of GABAergic neurons in the SCN. In addition, VWR exercise reduces the levels of BACE1 and GSK3β and decreases the APP and phosphor‐tau protein accumulation in the hypothalamus, indicating that exercise may directly affect the sleep–wake cycle pacemaker and regulate the function of the SCN and protect against AD. Exercise may serve as a behavioral therapy to be considered in clinical use to improve the circadian disturbances and alleviate the progression of AD. However, the current research has limitations. First of all, in the current study we used only male mice. In the future new study we plan to examine the sex differences of VWR exercise in the circadian rhythm disorder and AD‐related pathological changes in AD mice. Second, more comprehensive assessments of circadian rhythm characteristics and sleep‐related neurotransmitter changes at multiple time points are needed. Third, the molecular mechanisms underlying the beneficial effects of exercise on AD sleep disorders require further investigation, including a human cohort study to verify our findings.

## CONFLICT OF INTEREST STATEMENT

The authors declare that they have no conflicts of interest.

## CONSENT STATEMENT

Animal care was reviewed and the research protocal approved by the Institutional Animal Care Committee at Dalian Medical University.

## Supporting information



Supporting Information

Supporting Information
