# Supplementary material for: Voluntary wheel running exercise improves sleep disorder, circadian rhythm disturbance, and neuropathology in an animal model of Alzheimer's disease
Source: Alzheimers Dement. 2025 Jun 24;21(6):e70314. doi: 10.1002/alz.70314 (PMC12187974; doi:10.1002/alz.70314)
Supplement: Supplementary file 2 — Supporting Information [file ALZ-21-e70314-s001.docx]

**Supplementary material**

**Table 1. Antibodies used in this study.**

| **Antibody** | **Dilution** | **Application** | **Cat.Number** | **Company** |
| --- | --- | --- | --- | --- |
| Anti-BMAL1  Anti-BMAL1  Anti-KAT13D / CLOCK | 1: 1000  1:200  1:1000 | WB  IF  WB | ab3350  ab3350  ab3517 | Abcam  Abcam  Abcam |
| Anti-KAT13D / CLOCK | 1: 200 | IF | ab3517 | Abcam |
| Anti-ROR alpha | 1: 200 | IF | ab60134 | Abcam |
| Anti-Rev-erbα (RS-14) | 1: 200 | IF | Sc-100910 | Santa Cruz |
| Anti-β-Amyloid,1-16 (6E10) Anti-β-Amyloid,1-16 (6E10)  Anti-Phospho-Tau (Thr231) | 1: 1000  1: 2000  1: 1000 | IF  WB  WB | 803001  803001  MN1040 | Biolegend  Biolegend  ThermoFisher |
| Anti-Phospho-Tau (Thr231) | 1: 100 | IF | MN1040 | ThermoFisher |
| Anti-Iba1 | 1: 1000 | IF | 01127991 | Wako |
| Anti-GFAP | 1: 5000 | IF | ab7260 | Abcam |
| Anti-GAPDH (14C10)  Anti-VGAT | 1:4000  1:100 | WB  IF | 2118S  MA524643 | Cell signaling  ThermoFisher |
| Anti-Neurofilament heavy  Anti-GSK-3β (27C10)  Anti-BACE1 (D10E5) | 1:4000  1:1000  1:1000 | IF  WB  WB | ab8135  9315  5606 | Abcam  Cell signaling  Cell signaling |

**Table 2. List of primers used for quantitative real-time PCR assays.**

| **mRNA** | **Forward （5’-3’）** | **Reverse（5’-3’）** |
| --- | --- | --- |
| *Gapdh* | CTGTGCCCATCTACGAGGGCTAT | TTTGATGTCACGCACGATTTCC |
| *Clock* | CGGCGAGAACTTGGCATT | AGGAGTTGGGCTGTGATCA |
| *Bmal1* | CGCCTCTACCTGTTCAAAGAAAAA | TCACCCGTATTTCCCCGTTC |
| *Rev-erbα* | CTGCGGACCCTGAACAACAT | AATAGGGGAGGATGGGAGCAT |
| *Rev-erbβ* | CCAGAGCCCACAAGGATACC | GCATGGCCGTTTGGGTAATG |
| *Cry1* | CAGACTCACTCACTCAAGCAAGG | TCAGTTACTGCTCTGCCGCTGGAC |
| *Per1* | AAACGGCAAGCGGATGG | GCCATACAGTGGAGGACGAAA |
| *Rorα* | GCACCTGACCGAAGACGAAA | GAGCGATCCGCTGACATCA |
| *Rorβ* | GACCCACACCTACGAGGAAA | GTGATCTGGATGGCACACTG |
